# Supplementary material for: MetaRibo-Seq measures translation in microbiomes
Source: Nat Commun. 2020 Jun 29;11:3268. doi: 10.1038/s41467-020-17081-z (PMC7324362; doi:10.1038/s41467-020-17081-z)
Supplement: Supplementary file 10 — Supplementary Data 7 [file 41467_2020_17081_MOESM10_ESM.zip › File2/Confidence_High_Taxonomy/72013_out.krona.html]

Javascript must be enabled to view this page.

members
magnitude
magnitudeUnassigned
count
unassigned
taxon
rank

72013\_out

2

2759
superkingdom
2

2
kingdom
4751

1
phylum
1913638

subphylum
1
451827

2233521
class
1

78899
order
1

78905
family
1

78914
genus
1


SRS1041145\_contig\_number\_contig-100\_4486.188559
78915
1
species

1
subkingdom
451864

1
phylum
4890

147538
1
subphylum

1
class
147550

222544
subclass
1

SRS077392\_contig\_number\_4061
